# Antibody-Based In Vivo Imaging of Central Nervous System Targets—Evaluation of a Pretargeting Approach Utilizing a TCO-Conjugated Brain Shuttle Antibody and Radiolabeled Tetrazines

**DOI:** 10.3390/ph15121445

**Published:** 2022-11-22

**Authors:** Christoph Bredack, Martin R. Edelmann, Edilio Borroni, Luca C. Gobbi, Michael Honer

**Affiliations:** 1 Roche Pharma Research and Early Development, Roche Innovation Center Basel, Neuroscience and Rare Diseases, Biomarkers and Translational Technologies, F. Hoffmann-La Roche Ltd., CH-4070 Basel, Switzerland; 2 Roche Pharma Research and Early Development, Roche Innovation Center Basel, Therapeutic Modalities, Small Molecule Research, Isotope Synthesis, F. Hoffmann-La Roche Ltd., CH-4070 Basel, Switzerland; 3 Department of Pharmacy and Pharmacology, University of Bath, Bath BA2 7AY, UK; 4 Roche Pharma Research and Early Development, Roche Innovation Center Basel, Therapeutic Modalities, Small Molecule Research, Medicinal Chemistry, F. Hoffmann-La Roche Ltd., CH-4070 Basel, Switzerland

**Keywords:** antibody, click chemistry, CNS, PET, pretargeting, tetrazine, trans-cyclooctene, tritium

## Abstract

Bioorthogonal pretargeted imaging using the inverse-electron-demand Diels–Alder (IEDDA) reaction between a tetrazine (Tz) and a *trans*-cyclooctene (TCO) represents an attractive strategy for molecular imaging via antibodies. The advantages of using a pretargeted imaging approach are on the one hand the possibility to achieve a high signal-to-noise ratio and imaging contrast; on the other hand, the method allows the uncoupling of the biological half-life of antibodies from the physical half-life of short-lived radionuclides. A brain-penetrating antibody (mAb) specific for β-amyloid (Aβ) plaques was functionalized with TCO moieties for pretargeted labeling of Aβ plaques in vitro, ex vivo, and in vivo by a tritium-labeled Tz. The overall aim was to explore the applicability of mAbs for brain imaging, using a preclinical model system. In vitro clicked mAb–TCO–Tz was able to pass the blood–brain barrier of transgenic PS2APP mice and specifically visualize Aβ plaques ex vivo. Further experiments showed that click reactivity of the mAb–TCO construct in vivo persisted up to 3 days after injection by labeling Aβ plaques ex vivo after incubation of brain sections with the Tz in vitro. An attempted in vivo click reaction between injected mAb–TCO and Tz did not lead to significant labeling of Aβ plaques, most probably due to unfavorable in vivo properties of the used Tz and a long half-life of the mAb–TCO in the blood stream. This study clearly demonstrates that pretargeted imaging of CNS targets via antibody-based click chemistry is a viable approach. Further experiments are warranted to optimize the balance between stability and reactivity of all reactants, particularly the Tz.

## 1. Introduction

Many antibodies are characterized by exquisite specificity and affinity towards their targets. This feature makes them ideal tracers for molecular imaging techniques, such as positron emission tomography (PET), particularly for targets for which a specific small molecule PET tracer does not exist. Antibodies are particularly stable molecules with long-lasting exposure in the bloodstream. Their long biological half-life does not match with the physical half-life of the typically used short-lived PET radioisotopes, such as ^11^C (t_1/2_: 20.4 min) or ^18^F (t_1/2_: 109.7 min). The rapid decay of the label along with the slow pharmacokinetics of the antibody will not allow the selection of an appropriate imaging time point yielding a sufficient signal in brain tissue over a background signal in the bloodstream (limited imaging contrast). Consequently, antibodies would need to be labeled with longer-lived isotopes such as ^89^Zr (t_1/2_: 3.27 d) [1,2]. However, this would lead to an increased and possibly intolerable radiation exposure to patients [3,4,5,6].

Most of the challenges related to imaging strategies with directly radiolabeled antibodies can be addressed by a pretargeting approach where antibody and radiotracer administration are temporally uncoupled and the in situ labeling of a pretargeted antibody occurs at a time point when the modified antibody is still bound to the target antigen but already cleared from non-target tissues and the bloodstream. Such an approach increases the signal-to-noise ratio and generates sufficient contrast for imaging of a target protein. Furthermore, it decreases the radioactive burden to non-target tissues since shorter-lived PET nuclides can be used.

Various pretargeting concepts have been reported, including the use of bispecific antibodies binding to the antigen and a radiolabeled hapten based on the high streptavidin-biotin affinity [7,8] or oligonucleotide-modified antibodies combined with radioactively labeled complementary nucleic acid strands [9]. The bioorthogonal click chemistry reactions, another modality used for a pretargeting approach, have been extensively investigated, especially in preclinical oncology models [4,10,11,12,13,14,15]. The IEDDA reaction of a 1,2,4,5-tetrazine group and a *trans*-cyclooctene (TCO) derivative is of particular interest [16,17,18]. With reaction constants of up to 3.3 × 10^6^ M^−1^ s^−1^ [19], this reaction is rapid and biological processes are not influenced due to the selectivity and orthogonality under physiological conditions [20,21,22,23]. This click-reaction has been reported for fluorescent labeling of nanoparticles [24,25], antibodies [26,27], oligonucleotides [28,29], or the synthesis of radiopharmaceuticals [15,30,31,32].

The use of antibodies, such as imunoglobulin G (IgG), for pretargeted in vivo PET imaging of central nervous system (CNS) targets is severely limited by their large size, hydrophilicity, and the associated poor penetration of the blood-brain barrier (BBB) [33]. Usually only around 0.1% of the injected dose is able to enter the CNS by passive transport mechanisms [33,34]. Concepts have been developed to transport large molecules across the BBB more efficiently. One concept is the use of the transferrin receptor (TfR), which enables the active transport of molecules across the BBB via receptor-mediated transcytosis [35,36,37,38,39] due to its expression in endothelial cells of the BBB [40] and on neurons [41]. A Roche-developed brain-shuttle module was shown to have up to 55-fold increased uptake of an antibody-based cargo across the BBB into the brain [42,43].

This study aimed to establish an antibody-based pretargeting approach in combination with the IEDDA cycloaddition for in vivo imaging of targets expressed in the CNS. The pretargeting approach was chosen to achieve a high signal-to-noise ratio in the brain regions expressing the target compared to non-target regions. The amyloid-beta (Aβ) binding antibody mAb31 in its brain shuttle version (mAb31-BrainShuttle) along with the PS2APP Alzheimer’s disease mouse model was used as a tracer-target pair for this proof-of-concept study [44]. This animal model shows a very pronounced amyloidosis in broad parts of the brain, with only a small extent of pathology in the cerebellum, which can be used as a reference region for in vivo experiments. Furthermore, mAb31-BrainShuttle is a well-established antibody with specificity for Aβ plaques and with increased brain uptake compared to a classical IgG [42,43]. A tritiated version of a tetrazine derivative known from the literature [45] was synthesized in-house and was used in this study as a tool compound for in vitro, ex vivo, and in vivo experiments. Tritium, with its β-decay pattern leading to unmatched sensitivity and spatial resolution, was used in an ex vivo autoradiography readout. Along with its very long physical half-life (t_1/2_: 12.3 y), tritium was the obvious choice for our preclinical proof-of-concept study. The use of tritium permits the analysis of late time points after the injection of the tracer and allowed to explore the overall applicability of mAbs for the imaging of targets in the CNS.

## 2. Results

### 2.1. TCO Modification and Radiolabeling of the Antibody Construct

**Figure 1:**
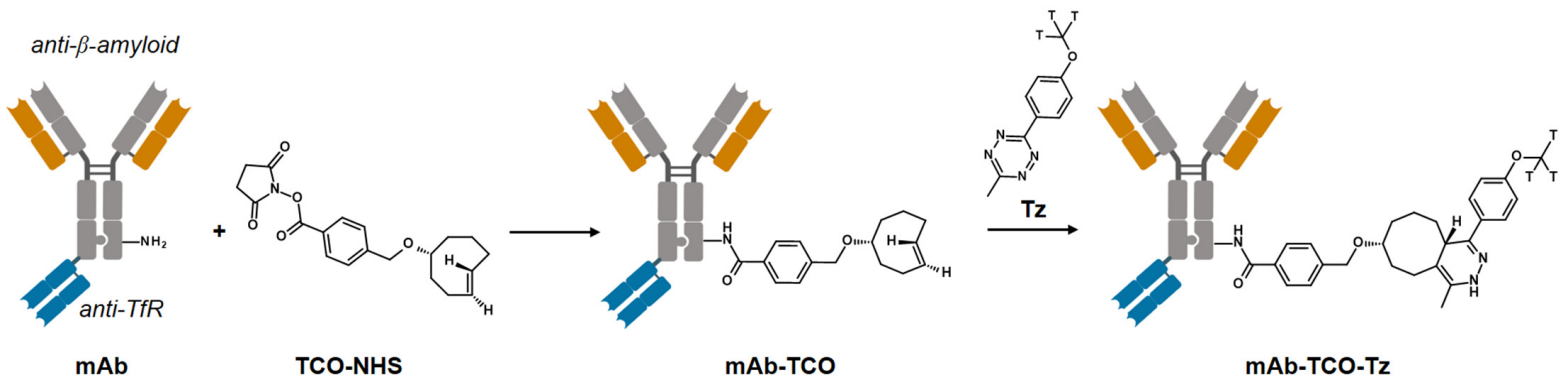
Nomenclature of compounds used in this study. Conjugation of NHS–TCO to mAb and the IEDDA cycloaddition between the modified antibody mAb–TCO and a [^3^H]-labeled tetrazine derivative Tz to form mAb–TCO–Tz.

The mAb was conjugated with TCO–NHS reagent to obtain mAb–TCO for subsequent click chemistry experiments (Figure 1). In order to determine the degree of labeling, the TCO-modified antibody was analyzed by LC-MS after reductive separation of the chains. The MS data revealed an average of 2.6 TCO moieties per antibody molecule (Supplemental Figures S1–S3 and Supplemental Tables S1–S3).

**Figure 2:**
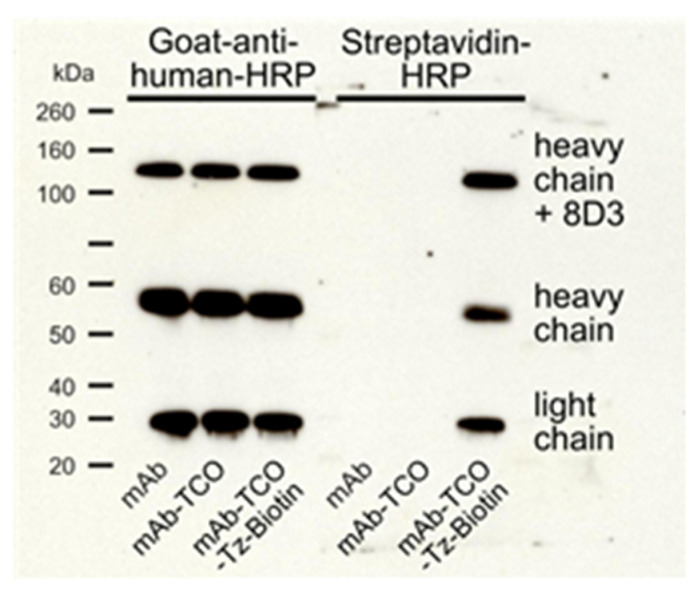
Western blot analysis of the mAb, mAb–TCO and mAb–TCO–Tz–biotin, generating three distinct bands, did not reveal a difference in the overall biochemical composition (Goat-anti-human-HRP detection). A TCO labeling of all three antibody chains was detected (Streptavidin-HRP detection).

The click chemistry reactivity of mAb–TCO was tested with tetrazine–biotin (Tz–biotin). SDS-PAGE under reducing conditions, followed by a Western Blot analysis of mAb, mAb–TCO, and mAb–TCO–Tz–biotin showed that the overall structure was not affected after modifications, no proteolytic fragments were detected. Three distinct bands of the antibody construct were detected, corresponding to the light (~25 kDa), heavy (~50 kDa), and heavy + BrainShuttle (~100 kDa) chains. The click reaction with the Tz–biotin confirmed that all three chains were labeled with TCO (Figure 2).

In this study, a tritiated tetrazine (Tz) was used in various experiments to radiolabel the mAb via click chemistry: (1) For tritium labeling of mAb–TCO to receive mAb–TCO–Tz (Figure 1); (2) in vitro click reaction on mouse brain sections with pre-incubated mAb–TCO and tritiated Tz; (3) for ex vivo (mAb–TCO injected in vivo, Tz incubation ex vivo on brain sections); and (4) in vivo pretargeting (mAb–TCO injected in vivo, Tz applied in vivo) experiments.

mAb–TCO was clicked in a 1:1 molar ratio with the Tz in vitro in order to receive the radiolabeled antibody construct mAb–TCO–Tz. An average radiochemical yield of 51 ± 13% (*n* = 5) was achieved. Furthermore, a specific activity of 8 ± 2 kBq/µg, which is equal to a molar activity of 1.7 ± 0.4 TBq/mmol, was achieved.

### 2.2. Retained Antibody Binding to Aβ Plaques after TCO modification

**Figure 3:**
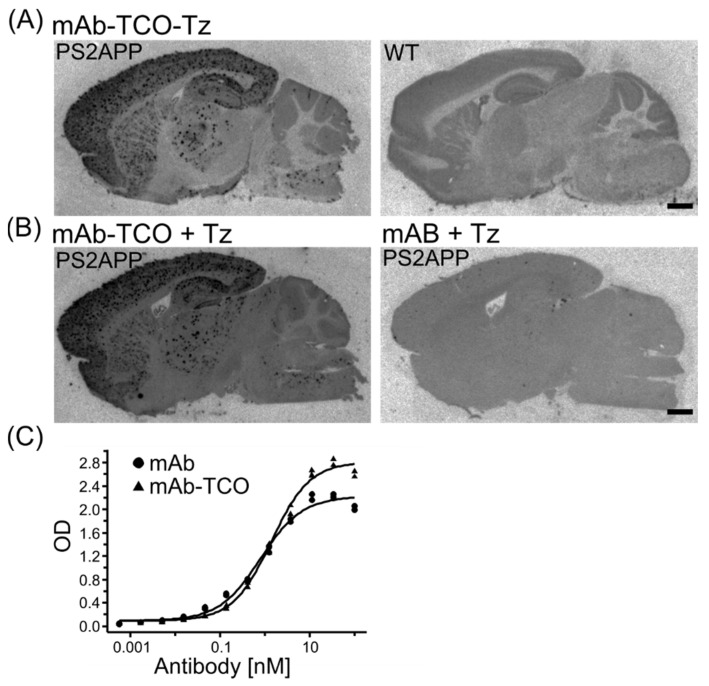
Maintained mAb affinity towards Aβ plaques after TCO conjugation. (**A**) In vitro autoradiography of mAb–TCO–Tz (clicked in vitro in a vial before incubation) showing binding to Aβ plaques in PS2APP mouse brain sections in comparison to WT brain sections. Scale = 1 mm. (**B**) In vitro autoradiography of mAb–TCO after in vitro, on slide click reaction with Tz showing a clear signal of Aβ plaque binding in PS2APP mouse brain sections. Incubation of mAb (lacking the TCO) followed by in vitro, on-slide click reaction using Tz on transgenic mouse brain sections (as a control) did not yield a specific Aβ plaque-related signal, revealing the high selectivity and specificity of the click reaction. Scale bar = 1 mm. (**C**) Comparison of mAb vs. mAb–TCO binding to Aβ. The applied ELISA assay did not reveal a change in the binding affinity towards the antigen.

The introduction of TCO groups and subsequent click reaction may influence the binding characteristics of the antibody towards its Aβ plaque epitope. To assess a potential impact by the modification, the TCO-modified mAb was clicked in vitro with the Tz, yielding mAb–TCO–Tz. Incubation of brain sections using the tritium-labeled mAb–TCO–Tz revealed a clear difference in binding pattern between transgenic (PS2APP) animals and wildtype (WT) controls (Figure 3A). The radiolabeled antibody specifically visualized Aβ plaques on brain sections from transgenic animals in reported brain regions, such as the cortex, hippocampus, or thalamus [44]. No specific signals on WT brain sections were observed. The binding ratios in the target region of interest (transgenic vs. WT) were above two in all cases, which confirms the preserved specificity of the antibody against Aβ plaques.

In order to mimic a pretargeting experiment, PS2APP brain sections were first incubated in vitro with mAb–TCO, followed by an on-slide click reaction with Tz. Autoradiographic analysis of the brain slices revealed clear imaging of Aβ plaques (Figure 3B). As a negative control, brain sections incubated with TCO-unconjugated mAb did not show any significant radioactive signal with a low background. The small molecule Tz led to a slightly higher background in non-target tissue in vitro due to non-specific binding. However, binding ratios in the cerebellum, cortex, and hippocampus of mAb–TCO-treated vs. mAb-treated brain sections were 3.90, 6.66, and 4.56, respectively. This result confirms that TCO modification of mAb does not affect the specificity towards Aβ plaques, but also highlights the exquisite specificity of the click reaction between TCO and Tz in vitro on a brain section.

Further in vitro assays were performed to confirm the retained functionality of the modified mAb. The binding affinity of mAb–TCO towards Aβ was tested by ELISA. With an EC_50_ of 1.4 nM for mAb–TCO compared to an EC_50_ of 0.8 nM for the non-conjugated mAb (Figure 3C), no pronounced impact on binding affinity was observed in a single experiment. This observation was confirmed by in vitro immunohistochemistry experiments using mAb and mAb–TCO on PS2APP brain sections (see Supplemental Figure S4).

### 2.3. TCO Modified Antibody Retains Binding to Transferrin Receptor and Enters the Brain

**Figure 4:**
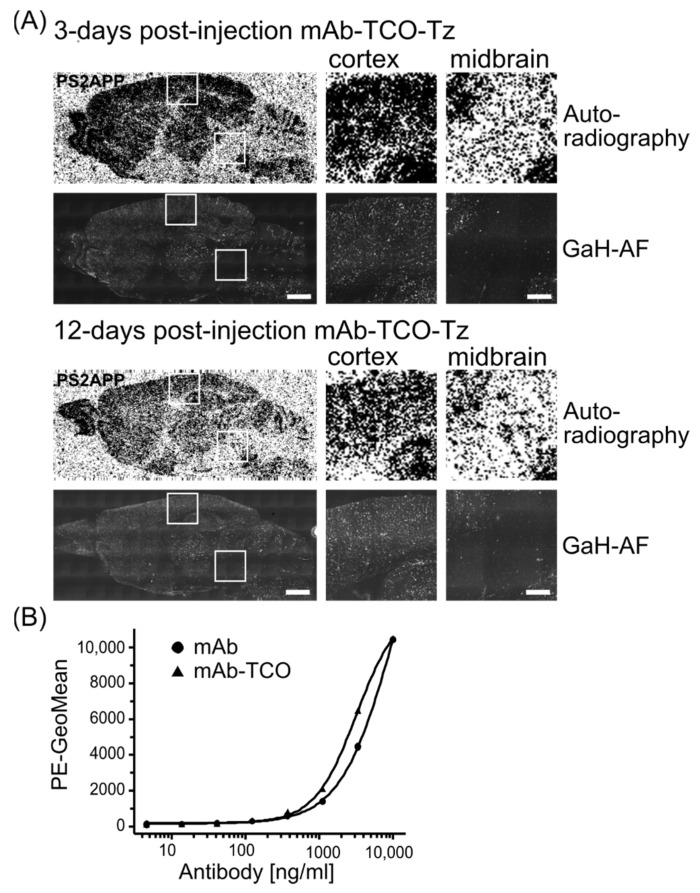
Maintained TfR binding and brain uptake of mAb–TCO–Tz. (**A**) Autoradiography of brain sections from PS2APP animals, 3-days and 12-days post-injection of mAb–TCO–Tz in vivo, showed a clear signal in Aβ-plaque-containing regions. Colocalization between the radioactive signal (autoradiography) and the mAb (GaH-AF) was visualized, using immunohistochemistry stainings, in the cortex, hippocampus, thalamus, and other Aβ-containing brain regions. Scale = 1 mm, magnification scale = 200 μm. (**B**) For completeness, a FACS analysis proved a slight influence of the TCO coupling on the mAb binding (mAb–TCO, triangle) to the TfR as compared to the non-modified mAb (circle).

The binding of the antibody to the transferrin receptor (TfR) is crucial for a sufficient brain uptake of the modified brain shuttle antibody. In vivo experiments were conducted to assess TfR binding and the ability of the antibody construct to enter the brain after TCO modification. Upon intravenous (IV) injection of mAb–TCO–Tz (19.1 ± 1.3 µg/g, 125.8 ± 40.9 kBq/g, 3.4 ± 1.1 MBq) in PS2APP mice, brain uptake and target binding were analyzed using autoradiography and immunofluorescent staining. In Aβ-deposit-containing brain regions, a radioactive signal was detected with high specificity and contrast. It colocalized with the presence of the antibody, as determined by immunofluorescent staining against the injected modified mAb–TCO–Tz (Figure 4A). Plaque-free brain regions did not show a signal in both analysis modalities. High brain uptake of mAb–TCO–Tz was corroborated by a quantitative tissue sampling and counting analysis yielding %ID/g values of 0.51 in the cortex vs. 0.33 in the cerebellum (low plaque density) after 3 days. Uptake values decreased to 0.21 %ID/g in the cortex and 0.09 %ID/g in the cerebellum 12 days post-injection of the antibody (detailed biodistribution data see Supplemental Table S4).

The binding affinity of mAb–TCO towards TfR was assessed in vitro using Fluorescence-Activated Cell Sorting (FACS) flow cytometry analysis. A strong influence of TCO-conjugation on the binding characteristics towards TfR was not detected. The EC_50_ for mAb–TCO was 3.19 ng/mL whereas the EC_50_ of the conjugated mAb was 10.16 ng/mL (Figure 4B).

### 2.4. Conjugated TCO Remains Reactive towards Tz after In Vivo Injection

**Figure 5:**
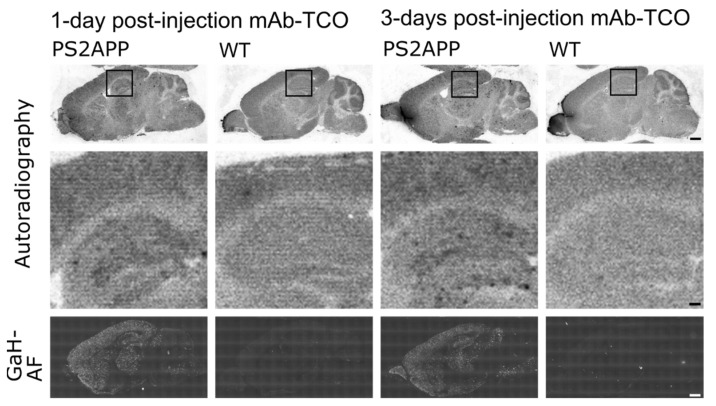
Maintained reactivity of the TCO moiety in vivo. Autoradiography results of mouse brain sections from PS2APP and WT controls following ex vivo click-reaction using the Tz, 1 day and 3 days post-injection of mAb–TCO (top row, scale bar = 1 mm). The magnification shows a clear, autoradiographic signal in brain sections of the transgenic animal and not the WT (middle row, scale = 200 μm). Immunohistochemical staining (GaH-AF) proves mAb–TCO enters the brain and is responsible for the detected signal (bottom row, scale bar = 1 mm).

After confirming the preserved affinities of mAb–TCO in in vitro and in vivo experiments, the interval between antibody injection and radiotracer application, i. e., the optimal pretargeting interval and the persistence of TCO functionality over time, were analyzed. Upon treatment of PS2APP and WT control animals with 20 mg/kg mAb–TCO, animals were sacrificed after four different time points (1-, 3-, 6-, and 12-days post-injection), and their brains dissected. As anticipated, the TCO-modified antibody passed the blood–brain barrier and bound to Aβ plaques in the expected brain regions (including cortex, hippocampus, and thalamus), as demonstrated by immunofluorescent detection of the mAb–TCO antibody (Figure 5). The mAb–TCO construct entered the brain in a comparable manner as the unconjugated mAb (comparison see Supplemental Figure S5). Brain sections were incubated with the Tz in order to test the reactivity of the TCO moiety by an ex vivo, on slide click reaction. Six- and twelve-days post-injection, no specific difference between the transgenic and the WT animals was detected (data not shown). In contrast, the earlier time points 1 day and 3 days post-injection of mAb–TCO revealed a specific signal in the autoradiographic detection on brain sections of the transgenic animals. Aβ plaques were clearly visualized on PS2APP mouse brain sections in areas such as the cortex, hippocampus, thalamus, pons, and colliculi, while brain sections of WT control animals did not show the characteristic dotted binding pattern (Figure 5). Radioactive signals on WT mouse brain sections only represented non-specific background binding (Table 1). Consequently, the reactivity of the TCO moiety of the injected mAb is limited to a pretargeting time interval of 3 days to ensure a successful in vivo click reaction and a sufficient radioactive signal.

### 2.5. In Vivo Click Reaction Did Not Reveal a Specific Radioactive Signal in the Brain

**Figure 6:**
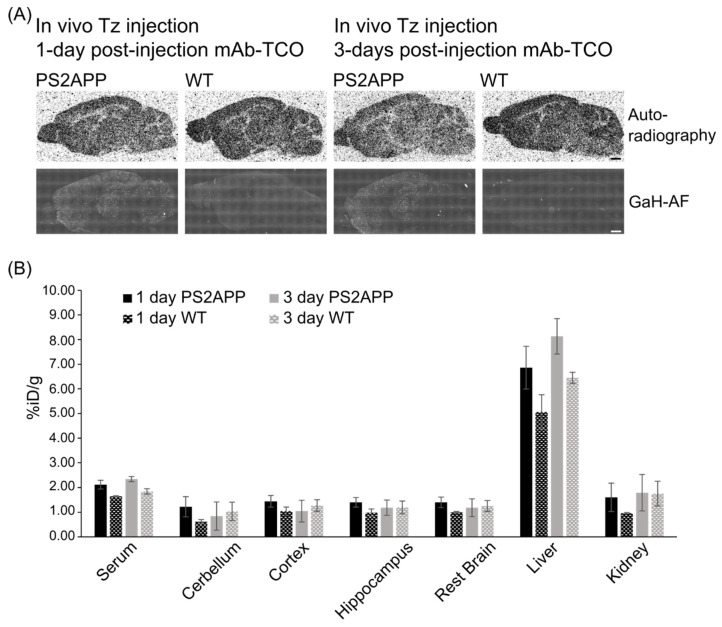
In vivo click reaction did not reveal a specific Aβ plaque signal. (**A**) Tz was applied in vivo 1 day and 3 days post-injection of the mAb–TCO in PS2APP animals and WT controls at day 0. The in vivo click reaction between the Tz and the bound mAb–TCO did not show a specific signal, which could be detected by autoradiography in neither the transgenic animals nor the control WT animals. The mAb entered the brain and bound to Aβ plaques as revealed by the GaH-AF staining. Scale bar = 1 mm. (**B**) Biodistribution data of the injected animals showed a rather equal distribution of the radioactivity (expressed in %ID/g) throughout the genotypes and time points.

Finally, an in vivo click reaction between injected mAb–TCO and the Tz was attempted in PS2APP and WT animals. Animals were injected with 20 mg/kg mAb–TCO IV via the tail vein. Based on previous results described above, animals were injected with the Tz (123.5 ± 24.5 ng; 1.8 ± 0.4 MBq; 55.1 ± 4.8 kBq/g) 1 day and 3 days post-injection of mAb–TCO. The Tz entered the brain and the radioactive signal reached 1.4 ± 0.2% ID/g in the cortex 1 day after injection and 1.2 ± 0.4 %ID/g in the cerebral cortex of the transgenic animals 3 days after the antibody pretargeting and injection of the Tz (for further details see Figure 6B). However, no difference in binding pattern and signal intensity was observed by autoradiography in PS2APP animals compared to WT control animals. In particular, no Aβ plaque imaging was detected by autoradiography (Figure 6A). Image analysis showed similar uptake ratios in all regions of interest in transgenic animals compared to control animals. The presence of mAb–TCO on the brain sections was confirmed by immunofluorescent staining (Figure 6A), but the in vivo click reaction of mAb–TCO with the radioactive Tz only occurred to an insufficient degree. Furthermore, the biodistribution data demonstrated a rather homogenous distribution of the radioactive signal throughout the genotypes and time points. The tissue counting results confirmed the uniform distribution of the imaging experiments (Figure 6B).

## 3. Discussion

The described study combines an antibody-based pretargeting approach with the bioorthogonal click reaction between a strained *trans*-cyclcooctene and an electron-deficient tetrazine by IEDDA. This concept has been extensively studied for the imaging of peripheral targets, for example, targets overexpressed in a xenograft-based oncology mouse model [4,10,12,46,47]. It was demonstrated that in vivo PET imaging is possible with high tracer uptake in tumors, a high tumor to non-target tissue ratio, and a significantly decreased radioactive burden to non-tumor tissues. However, all reported studies about pretargeted imaging were limited to peripheral targets. The presented work is among the first studies applying the antibody-based pretargeting approach to a target in the CNS and attempting to exploit all the advantages of the described concept.

TCO-modified mAb (mAb–TCO) was successfully clicked in vitro with tritium-labeled tetrazine (Tz) yielding mAb–TCO–Tz. Brain sections, incubated in vitro with mAb–TCO–Tz, showed clear plaque imaging in PS2APP transgenic mouse brain sections, whereas no specific signal was detected in WT controls. Furthermore, an in vitro click reaction on brain sections with pretargeted mAb–TCO and Tz as the secondary agent confirmed the reactivity on transgenic PS2APP brain tissue. In contrast, the same brain sections incubated with mAb (without TCO) and subsequent Tz treatment did not show any specific radioactive signal. This finding confirmed that the binding of the modified mAb to the Aβ plaques and the accessibility of the TCO moieties on the antibody for Tz is maintained in vitro. A slightly higher background originating from the Tz was observed on the PS2APP and WT brain sections compared to an incubation with mAb–TCO–Tz. However, similar to most small molecules, the Tz shows some degree of non-specific binding to tissue constituents in brain sections such as lipids and membrane bilayers. Non-specific binding is for example influenced by the logD value, the polar surface area, and other physicochemical properties of the molecule. The in vivo behavior of the tracer cannot be predicted purely based on in vitro experiments.

Based on the results, in vivo experiments demonstrated the brain shuttling capability of in vitro-prepared mAb–TCO–Tz in PS2APP transgenic mice by autoradiography imaging and fluorescent staining. Colocalized signals of both autoradiographical imaging and immunofluorescent staining were detected in typical Aβ-containing brain regions. In a next step, the optimized pretargeting interval, the time between antibody injection and injection of Tz, was determined in PS2APP and WT control mice treated with mAb–TCO. After 1, 3, 6, and 12 days, the animals were sacrificed, the brain dissected, and the sections incubated with a tritiated Tz for an ex vivo click reaction. After 1-day and 3-days post-injection, a clear signal was detected that distinguished PS2APP transgenic from WT animals. Aβ deposits were visualized by autoradiography on transgenic animal brain sections only. The signal detected on WT sections represented background binding. The findings clearly demonstrated that a sufficient amount of antibody enters the brain as expected for proper visualization of the target. Furthermore, the conjugated TCO group remained chemically reactive to click with the applied tritiated Tz. Longer pretargeting intervals (6 days and 12 days), however, did not result in autoradiography signals, although mAb–TCO entered the brain, which was visualized by fluorescent detection using a secondary antibody. No signals by ex vivo click Tz treatment were observed after 6 days suggesting that the TCO moiety did not remain reactive long enough for an efficient click reaction. The TCO is likely to be inactivated by copper-catalyzed *trans*-*cis* isomerization. The *cis* isomer of TCO is orders of magnitude less reactive than the *trans* isomer [48], which might explain the lack of a signal 6 days and later after injection of the antibody. All in vitro and ex vivo click experiments suggested that the mAb–TCO passed the blood-brain barrier and bound to Aβ targets, and TCO remained reactive towards the Tz up to 3 days. Comparison of the binding ratios of an in vitro click reaction (incubation of PS2APP brain sections with mAb–TCO + Tz) with the ex vivo click reaction (in vivo injection of mAb–TCO followed by ex vivo Tz application on brain section) suggested that the reactivity of the TCO diminished after exposure to the in vivo metabolism in animals (see Supplemental Table S5). TCO became inactivated to a certain degree in vivo, less TCO was available for the click reaction, and the specific radioactive signal was consequently weaker.

In the next step, an in vivo click experiment was carried out using PS2APP transgenic and WT mice. First, a dose of 20 mg/kg mAb–TCO was injected followed by Tz injection 1 day and 3 days later. Two hours after the application of the Tz, the animals were sacrificed and analyzed for the presence of a genotype-specific brain signal. Even though the antibody and the Tz (Tz-only biodistribution data for comparison, see supplemental material Table S6) entered the brain as expected, no clear signal of target visualization in PS2APP transgenic mice was detected. Transgenic and WT brain sections looked similar, revealing a homogenous background for both time points analyzed. This finding indicated that the tracer entered the brain, but no in vivo click reaction took place or was detectable due to a high background signal. It remains to be analyzed why the click reaction did not occur to a sufficient amount in vivo after 1 day and 3 days, even though the previous experiments indicated 1 day and 3 days to be an optimal pretargeting time point, and several reasons can be considered. It has been reported that the strained ring of *trans*-cyclooctene may undergo *trans-cis* isomerization, thereby reducing its reactivity towards tetrazines dramatically [12,48]. Regarding the degree of labeling of the antibody with the TCO, “the more the better” could be considered. However, there is a tradeoff of potentially disturbed antibody functionality or pharmacokinetic characteristics when the conjugation degree with TCOs is too high [49]. The more TCO groups attached to the antibody, the more likely the functionality of binding to its target and the brain shuttling via the TfR are affected. An approach to increase the modification rate of the antibody, and minimizing the effect on the antibody functionality is to introduce PEG linkers between the antibody and the TCO moiety. The further away the TCO is from the antibody molecule, the lesser the influence of potential hydrophobic interactions between the TCO and the mAb, which can influence the binding behavior. Furthermore, steric hindrances are reduced, which could influence the access of the TCO to the lysine residues during the modification. However, the further a TCO protrudes from the antibody, the more exposure to the surrounding, which can increase *trans*-*cis* isomerization [50]. Further approaches to increase the TCO loading of a mAb are the use of multi-functionalized moieties that can be attached to an antibody (like branched, dendritic scaffolds containing higher numbers of TCO molecules) [51,52].

In addition, it has not yet been demonstrated whether the Tz reaches the brain in sufficient quantities. Although the tracer was shown to penetrate into the brain (Supplemental Table S6), the concentration may have been too low. The ratio between the available Tz and the functional TCO might not be optimal for a detectable click reaction to occur. The biodistribution data of the Tz indicated a longer presence of the radioactive signal in the blood. The radioactive signal in the blood stream can potentially obscure the specific signal originating from a click reaction between the Tz and the bound antibody. Another reason for the failure of the in vivo click reaction could be the instability of the Tz. Tetrazines are highly reactive molecules and might degrade faster than expected in an in vivo animal system [53]. The metabolism of the organism could be responsible for the biotransformation to an inactive species lacking a reactive tetrazine group. For this early preclinical proof-of-concept study, a tritiated Tz was used as a model compound, which cannot be applied for PET studies. For future PET studies, radionuclides such as ^18^F or ^11^C are needed for Tz labeling. Those molecules may present a different radiometabolic pattern, as the radiolabel will be in a different location in the tetrazine compared to the ^3^H. This stability pattern needs to be specifically analyzed. Recently, it was attempted to reverse the approach, i. e., functionalizing the antibody with tetrazine and applying a radioactive TCO as a secondary agent for the bioorthogonal click reaction [53,54]. However, this approach seemed to be counterproductive in the case of a bioorthogonal labeling of brain targets using a click reaction, as the tetrazine is a highly reactive and unstable molecule. The longer a tetrazine remains bound to the target, the higher the risk for a decomposition of the tetrazine. Furthermore, the LogD values (as a parameter of lipophilicity), which are crucial for brain penetration, are higher for the TCO derivatives than for the tetrazine derivatives. A higher lipophilicity suggests an increased non-specific binding to plasma proteins and a faster oxidative metabolism and clearance of the compound, which may lead to a decreased brain uptake [55,56].

Furthermore, biodistribution data of Tz, mAb–TCO (t_1/2_ of around 2.4 days in blood plasma), mAb–TCO–Tz, and mAb–TCO + Tz unexpectedly indicated a longer half-life of the antibody construct and radioactive signal in the periphery including blood plasma, kidney, and liver (major excretion routes of antibodies). The antibody entered the brain and persisted for up to twelve days (longest time point analyzed). However, the antibody remained in the blood, as well, which could capture in vivo injected Tz. On the one hand, the prolonged retention of mAb–TCO, Tz, and consequently mAb–TCO–Tz led to a higher background signal in the blood pool. On the other hand, it decreased the level of Tz to penetrate the brain for a click reaction at the mAb–TCO-decorated Aβ plaques. Taken together, these processes could obscure a brain-specific radioactive signal and may have led to a failure to observe a signal of an in vivo click reaction in Aβ plaque pathology containing brain regions. One option to further improve the signal-to-noise ratio and thus the imaging contrast is the use of peripheral clearing agents. Those molecules would be injected after the mAb–TCO injection in vivo and are designed to be impenetrable to the CNS and capture TCO-modified antibodies in the periphery for rapid hepatic clearance (e.g., by modifying the clearing agent with galactose moieties). Consequently, mAb–TCO is removed from the blood, injected Tz is not captured in the blood, the background is reduced, and more Tz could penetrate into the brain for a click reaction at mAb–TCO- decorated Aβ plaques [46,57].

Despite the failed in vivo click reaction, the used approach represents a promising avenue for in vivo imaging of CNS targets via PET. The pretargeting approach is versatile and modular to be broadly applied to a number of antibodies and CNS targets, which currently cannot be approached by small molecule PET tracers. The latest generation of bifunctional antibodies allows an increased uptake of the pretargeting compound in the brain for a reliable detection [58,59]. Further experimental work needs to be performed to identify a TCO/tetrazine combination with increased stability paired with high reactivity. An increased stability of both components, possibly combined with a blood–brain barrier non-penetrable clearing agent [46,60], might lead to successful pretargeting-based in vivo imaging of brain targets.

Nonetheless, this study demonstrates the promising approach of using antibody-based click chemistry for in vivo brain imaging and may extend the application to targets currently considered undruggable and not approachable by a traditional small-molecule PET tracer.

## 4. Materials and Methods

The antibody construct mAb31-BrainShuttle was produced in-house and consists of an immunoglobulin G subclass 1 monoclonal antibody (mAb31) directed against Aβ [61] and a brain shuttle construct fused to the C-terminus of the heavy chain as described previously [42,43]. The brain shuttle construct is a single-chain single Fab fragment of an anti-mouse Transferrin monoclonal antibody [42]. Hereinafter the mAb31-BrainShuttle construct is referred to as mAb.

Solvents and reagents were obtained from commercial sources (as indicated) and were used without further purification.

**Scheme 1:**
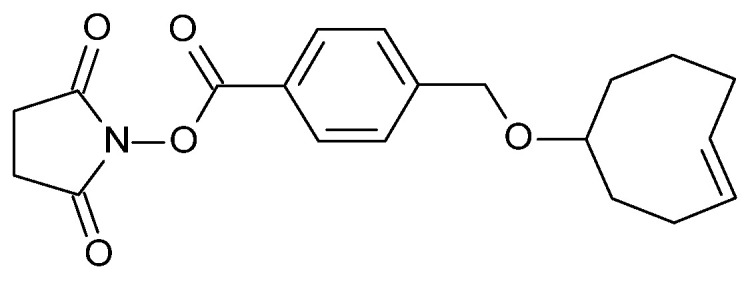
Dioxopyrrolidin-1-yl) 4-[[(4E)-cyclooct-4-en-1-yl]oxymethyl]benzoate (TCO–NHS).

Dioxopyrrolidin-1-yl) 4-[[(4E)-cyclooct-4-en-1-yl]oxymethyl]benzoate (TCO–NHS) (Scheme 1) was synthesized and prepared in-house following the method reported by Rossin et al. [10].

A colorless solid was isolated in a yield of 21% and purity of 99%. ^1^H NMR (300 MHz, CDCl_3_) δ 8.12 (d, *J* = 8.28 Hz, 2H), 7.51 (d, *J* = 8.28 Hz, 2H), 5.68 (m, 1H), 5.53 (m, 1H), 4.57 (m, 2H), 3.69 (br, dd, *J* = 9.99, 4.54 Hz, 1H), 2.91 (br, s, 4H), 2.38 (m, 2H), 2.24 (m, 2H), 2.07 (m, 1H), 1.84 (m, 3H), 1.54 (m, 1H), and 1.23 (m, 1H). LC-MS (ESI) *m*/*z* C_20_H_23_NO_5_ requires: 358.2 ([M + H]^+^).

**Scheme 2:**
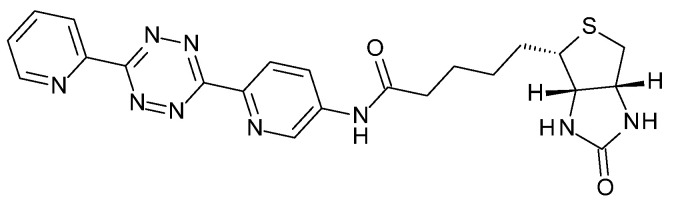
5-((3aS,4S,6aR)-2-Oxohexahydro-1H-thieno [3,4-d]imidazol-4-yl)-N-(6-(6-(pyridin-2-yl)-1,2,4,5-tetrazin-3-yl)pyridin-3-yl)pentanamide (Tz–biotin).

Biotin (217 mg, 870 µmol), 1-hydroxy-7-azabenzotriazole (148 mg, 1.04 mmol), and 1-ethyl-3-(3′-dimethylaminopropyl)carbodiimide hydrochloride (204 mg, 1.04 mmol) were dissolved in dry DMF (6 mL) under an argon atmosphere to give a colorless solution. Triethylamine (176 mg, 242 µL, 1.74 mmol) was added and the mixture was stirred at 22 °C for 1 h. Then, 6-(6-(pyridine-2-yl)-1,2,4,5-tetrazin-3-yl)pyridine-3-amine(230 mg, 870 µmol) was added and stirring was continued for 47 h at 50 °C. All volatiles were evaporated and the crude material was purified by flash chromatography (silica gel pretreated with 7% Et_3_N in heptane, 40 g column, 0% to 100% acetone in heptane in 20 min) to yield 86 mg (19%) of the desired compound Tz–biotin (Scheme 2).

^1^H NMR (600 MHz, DMSO-d_6_) δ 10.56 (s, 1H), 9.05 (d, *J* = 2.52 Hz, 1H), 8.94 (d, *J* = 4.69 Hz, 1H), 8.62 (d, *J* = 8.63 Hz, 1H), 8.59 (d, *J* = 7.74 Hz, 1H), 8.44 (dd, *J* = 2.52, 8.76 Hz, 1H), 8.16 (ddd, *J* = 1.81, 7.76, 7.76 Hz, 1H), 7.73 (ddd, *J* = 1.21, 4.68, 7.61 Hz, 1H), 6.45 (s, 1H), 6.37 (s, 1H), 4.29–4.34 (m, 1H), 4.12–4.17 (m, 1H), 3.07–3.20 (m, 1H), 3.05 (br s, 1H), 2.84 (dd, *J* = 5.14, 12.49, 1H), 2.52–2.61 (m, 1H), 2.39–2.48 (m, 1H), and 1.35–1.71 (m, 6H). LC-MS m/z C_22_H_23_N_9_O_2_S requires: 478.1 ([M + H]^+^), 500.3 ([M + Na]^+^).

To a 25 mL sealable tube, equipped with a stir bar, 452 mg (3.8 mmol) of 4-hydroxybenzonitrile, 1.9 mmol Zn(OTf)_2_, 188 mmol hydrazine monohydrate, and 2 mL (38 mmol) acetonitrile were added to give a purple-colored solution. The tube was sealed, and the reaction solution was stirred for 16 h at 80 °C. The reaction solution was cooled to 22 °C and transferred into a 250 mL three-neck round bottom flask. An amount of 25 mL of a 3.5 M sodium nitrate solution was added slowly with ice bath cooling, followed by dropwise addition of 1 M hydrogen chloride (75 mL) until no gas evolution was observed and a pH of 3–4 was reached. The mixture was extracted 3× with ethyl acetate, the combined organic phase was separated, dried over sodium sulfate, and concentrated in vacuo. The crude residue was purified using silica-based flash chromatography to yield 38% (271 mg, 1.44 mmol) of the desired phenol-precursor as an orange solid in a purity of 98%.

^1^H NMR (300 MHz, CDCl_3_) δ 8.52 (d, *J* = 8.88 Hz, 2H), 7.03 (d, *J* = 9.08 Hz, 2H), 5.21 (s, 1H), and 3.07 (s, 3H). LC-MS (ESI) *m*/*z* C_10_H_8_N_4_O requires: 189.4 ([M + H]^+^).

**Scheme 3:**
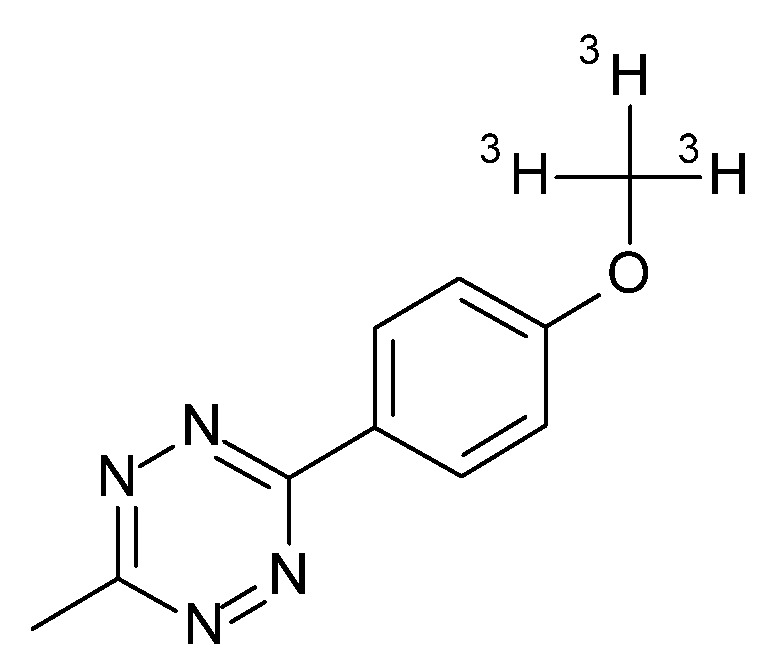
[^3^H]-3-(4-Methoxyphenyl)-6-methyl-1,2,4,5-tetrazine (Tz).

[^3^H]Methyl nosylate (1.85 GBq, 132 μg, 0.591 μmol), cesium carbonate (481 μg, 1.48 μmol), and 333 µg (1.77 µmol) of the phenol-precursor were dissolved in 400 μL tetrahydrofuran. The solution was stirred at 50 °C for 5 h. The reaction mixture was diluted with 1 mL dichloromethane and passed through a strong anion exchange (SAX) solid phase extraction (SPE) cartridge to remove the sulfonic acid by-product [62]. The solvent was removed and the residue dissolved in acetonitrile/water to purify the compound using preparative-HPLC to yield 278 MBq (15%) of Tz (Scheme 3) in a radiochemical purity of 98%. The specific activity of 3.3 TBq/mmol was taken from the starting compound [^3^H]methyl nosylate, since Tz did not ionize in mass spectrometric analysis and therefore no isotope pattern could be determined.

### 4.1. Antibody–TCO Conjugation

Ten-fold molar excess of TCO–NHS (10 mM in DMSO) was added to the mAb (4–5 mg/mL in PBS, pH 7.4). The solution was shaken orbitally for 2 h at 22 °C and purified using Slide-A-Lyzer Dialysis Cassettes (10,000 MWCO, Thermo Fisher Scientific, Waltham, MA, USA) against PBS, pH 7.4. The buffer was changed after 30 min for 4 times in total to generate mAb–TCO in a yield of 83 ± 11% (*n* = 9) with a degree of label 2.6 on average.

### 4.2. In Vitro Click Reaction

mAb–TCO (5 mg/mL in PBS, pH 7.4) and Tz (3.26 TBq/mmol, 185 MBq/mL in ethanol) were mixed in a 1:1 molar ratio (2–10 μM) and incubated for 1 h at 22 °C, and purified by dialysis (Slide-A-Lyzer Dialysis, 10,000 MWCO, Thermo Fisher Scientific, Waltham, MA, USA) against PBS (pH 7.4) to obtain mAb–TCO–Tz in a radiochemical purity of > 95%.

In analogy, mAb–TCO was coupled with a biotinylated Tz derivative. For this click reaction, a 10-fold excess of Tz–biotin (5 mM in DMSO) was used to ensure complete conversion to mAb–TCO–Tz–biotin.

### 4.3. Intact Protein Mass Spectrometry (MS) Analysis

For protein MS analysis of mAb–TCO, the protein concentration was adjusted to 0.5 mg/mL with 0.1 M Tris pH 7.8. The samples were chromatographed on a Waters nanoAcquity system (Waters Corporation, Milford, MA, USA) using an Agilent, Poroshell column (SB-C8, 0.5 mm x 75 mm, 5 μm, 300 Å, injection volume of 8 μL; Agilent, Santa Clara, CA, USA) heated to 65 °C. Eluent A was 0.1% trifluoroacetic acid in water, and eluent B was 0.1% trifluoroacetic acid in acetonitrile. The following gradient for the separation was applied: initial: 10% B; 0.2 min: 10% B; 1 min: 20% B; 11 min: 60% B; 15 min: 90% B; 16 min: 90% B; 17 min: 10% B; and 20 min: 10% B, with a flow rate of 70 μL/min. The eluent was analyzed with a Waters LCT Premier XE mass spectrometer (Waters Corporation) using the electrospray method for ionization of the sample (ESI, positive, V mode; with a capillary voltage of 3.0 kV and a cone voltage of 200 V). The raw data were deconvoluted with the MaxEnt1 software (Waters Corporation) and displayed using standard software.

### 4.4. SDS-PAGE and Western Blot Analysis

Modified antibody constructs were subjected to SDS-PAGE and Western blot analysis. SDS-PAGE was performed using the XCell SureLock™ Mini-Cell Electrophoresis System (Invitrogen, Thermo Fisher Scientific, Waltham, MA, USA) under reducing conditions. Samples were diluted in NuPAGE LDS Sample buffer (4×, Thermo Fisher Scientific) and NuPAGE Sample Reducing Agent (10×, Thermo Fisher Scientific), heated at 70 °C for 10 min, and 1–2 μg in 20 μL were separated using a Novex NuPAGE 4–12% Bis-Tris Gel (Thermo Fisher Scientific) with a 1× NuPAGe MOPS-SDS running buffer (Thermo Fisher Scientific). Separated proteins were transferred to a nitrocellulose membrane using the iBlot Gel Transfer System (Thermo Fisher Scientific, 7 min, 23 V) in conjunction with Novex Mini iBlot Gel Transfer Stacks (Thermo Fisher Scientific). The membrane was blocked for 1 h at 22 °C with 5% TopBlock (LubioScience, Lucerne, Switzerland), 0.1% Tween-20 in 1× PBS pH 7.6. mAb–TCO was detected using a goat-anti-human-horseradish peroxidase (HAP) coupled antibody (1:250,000, 1 h at 22 °C in 1% TopBlock/TBS/T, Pierce Thermo Fisher Scientific) and mAb–TCO–Tz–biotin was detected using a Streptavidin-HPA (1:200,000, 20 min, 22 °C in 5% TopBlock/TBS/T, Thermo Fisher Scientific). Membrane was rinsed twice in blocking buffer, washed 1 × 15 min followed by 2 × 5 min in blocking buffer, 1 × 5 min in TBS-T (1 × TBS pH 7.6, 0.1% Tween-20), and 1 × 5 min in TBS pH 7.6. The modified proteins were detected using Amersham ECL Western Blotting Detection Reagents (GE Healthcare Life Sciences, now Cytiva, Danaher Corporation, Little Chalfont, UK) and developed by applying standard procedures using Amersham Hyperfilm high performance chemiluminescence film.

### 4.5. Aβ ELISA Affinity Assay

Binding of fusion constructs to fibrillary Aβ was measured by an ELISA assay. Briefly, the 40 amino acid residue peptide Aβ(1–40) (Roche, Penzberg, Germany) was coated at 7 µg/mL in PBS onto Maxisorp plates (Nunc, Thermo Fisher Scientific, Waltham, MA, USA) for 3 days at 37 °C to produce fibrillary Aβ then dried for 3 h at 22 °C. The plate was blocked with 1% Crotein C and 0.1% RSA in PBS (blocking buffer) for 1 h at 22 °C, then washed once with wash buffer. Fusion polypeptides or controls were added at concentrations up to 100 nM in blocking buffer and incubated at 4 °C for 16 h. After 4 wash steps, constructs were detected by addition of anti-human-IgG-HRP (Jackson Immunoresearch, West Grove, PA, USA) at 1:10,000 dilution in blocking buffer (1 h, 22 °C), followed by 6 washes and incubation in TMB (Sigma Aldrich, St. Louis, MO, USA). Absorbance was read out at 450 nm after stopping color development with 1 M HCl.

### 4.6. Fluorescence-Activated Cell Sorting (FACS) Flow Cytometry Analysis

Binding of fusion constructs to murine transferrin receptor was tested by FACS analysis on mouse BA/F3 pro-B cells. Cells were harvested by centrifugation and washed once with PBS, and 5 × 10^4^ cells were incubated with a 1.5 pM to 10 nM dilution series of the antibody fusions in 100 µL RPMI/10% FCS for 1.5 h on ice. After 2 washes with RPMI/10% FCS, cells were incubated with goat-anti-human IgG coupled to Phycoerythrin (Jackson Immunoresearch, West Grove, PA, USA) at a dilution of 1:600 in RPMI/10% FCS for 1.5 h on ice. Cells were washed again, re-suspended in RPMI/10% FCS, and phycoerythrin fluorescence was measured on a FACS-Canto instrument (Becton-Dickinson Biosciences, Franklin Lakes, NJ, USA).

### 4.7. In Vivo Mouse Experiments

All animal experiments were performed according to the Swiss federal regulations on animal protection and to the rules of the Association for Assessment and Accreditation of Laboratory Animal Care International (AAALAC).

Adult male or female APP751 Swedish × PS2N141I transgenic (PS2APP) [44] mice on a homozygous C57BL/6 genetic background and C57BL/6 WT control animals at an age of 12–18 months, used for the experiments, were bred at the animal facility of Roche, Basel. The mice were group-housed in open cages and maintained on a 12:12-h light:dark cycle, with constant temperature (21–24 °C) and humidity (40–80%). Each cage was provided with unrestricted access to municipal water and sterilized food (Provimi Kliba 3436, Kaiseraugst, Switzerland). All cages were supplied with autoclaved sawdust bedding and environmental enrichments, which were applied to best practice animals’ welfare standards and rotated weekly. The mice were acclimated for at least 1 week before the start of the study. The study design is described in Table 2.

### 4.8. In Vivo Target Engagement

Animals were injected intravenously (IV) with 20 mg/kg mAb–TCO–Tz in PBS (injection volume 5 mL/kg, protein concentration 3–5 mg/mL) and sacrificed 3 days and 12 days post-injection. The brain was dissected and hemisected along the midline. One hemisphere was used for cryosections and immediately frozen on powdered dry ice. The other hemisphere was dissected to obtain brain regions including the cerebellum, hippocampus, cortex, and the rest brain, used for beta scintillation counting. The brain tissue was stored at −80 °C.

### 4.9. mAb–TCO Administration for Ex Vivo Click Reaction

Animals were injected intravenously (IV) with 20 mg/kg mAb–TCO in PBS (injection volume 5 mL/kg, protein concentration 3–5 mg/mL). After 1, 3, 6, 12 d post-injection, animals were sacrificed by decapitation. The brain was hemisected and frozen in powdered dry ice. The brain tissue was stored at -80 °C.

### 4.10. In Vivo Click Reaction

Animals were injected IV with a single dose of 20 mg/kg mAb–TCO. At 24 h and 72 h post-injection of the antibody, the animals received a single dose IV injection of 55.5 MBq/kg Tz in PBS, pH 7.4 (2.96 TBq/mmol, 9.25 MBq/mL, 5 mL/kg injection volume). At 2 h after the Tz injection, the animals were sacrificed by decapitation and plasma samples were collected. The brain was hemisected. One hemisphere was frozen on powdered dry ice, the other one was dissected, and brain regions including cerebellum, cortex, hippocampus, and rest brain were frozen on dry ice. The brain tissue was stored at -80 °C.

### 4.11. Immunohistochemistry, Radio-Immunohistochemistry

Animals were deeply anaesthetized using 3–5% (vol %) isoflurane in an anesthesia box with the support gas, oxygen/air (2:1). Animals were sacrificed by decapitation. The brains were removed, rinsed in 0.9% NaCl solution, blotted dry, hemisected along the midsagittal plane, and immediately frozen on powdered dry ice for histology and/or autoradiography.

Brains were cut sagittally on a cryostat (Leica CM 3050, Nussloch, Germany). Sections (10 µm thickness) were thaw-mounted on HistoBond plus glass slides (Marienfeld, Lauda-Königshofen, Germany), air dried for at least 2 h, and stored at −80 °C until further use.

Slides were defrosted, rehydrated with PBS (Thermo Fisher Scientific, Waltham, MA, USA), fixed with acetone (3 min, −20 °C, Sigma Aldrich, St. Louis, MO, USA) and blocked for 20 min at 22 °C (1% bovine serum albumin + 1% ovalbumin + 1% normal goat serum in PBS).

The mAb constructs, either applied in vitro or injected in vivo, were detected using a fluorescently labeled secondary antibody (goat-anti-human Alexa Fluor (AF) 488, AF 555, or AF 647, 15 µg/mL in 1% normal goat serum in PBS, 1 h, 22 °C, Thermo Fisher Scientific). The sections were counterstained with 1 μg/mL 4′,6-diamidino-2-phenylindole (DAPI, Roche, Switzerland) in PBS for 3 min. Sections were thoroughly washed with PBS between each incubation step. Standard SudanBlack B (0.3% in 70% ethanol, 3 min, Merck KgaA, Darmstadt, Germany) staining was used to quench lipofuscin autofluoresence. Microscopic, whole-slice images were acquired using the Metafer 4 slide scanner (MetaSystems, Altlussheim, Germany).

Fixed and blocked brain sections from transgenic and wild-type animals were incubated with 1 μg/mL mAb–TCO–Tz antibody solution in 1% normal goat serum in PBS for 1 h at 22 °C or 4 °C for 16 h. After intensive washing with PBS, the sections were air- dried at 4 °C and used for autoradiography.

For the in vitro and ex vivo on-slide click reaction, the brain sections of transgenic animals were defrosted, re-hydrated and blocked (no blocking step for ex vivo on-slide click reaction) as described above. In vitro staining with 1 μg/mL mAb–TCO or mAb as control in 1% normal goat serum in PBS, 4 °C for 16 h, was carried out. Slides were washed for 3 × 5 min with PBS followed by 3 × 5 min with a Ringer solution (146.5 mM NaCl, 2.7 mM KCl, 0.85 mM MgCl_2_, 1.2 mM CaCl_2_, 1.2 mM Na_2_HPO_4_, 0.27 mM NaH_2_PO_4_, pH 7.4; Merck KGaA, Fluka, Darmstadt, Germany).

The on-slide click-reaction was carried out for 2 h at 22 °C using 100 µL to 200 µL of 100 nM Tz (2.96 TBq/mmol, 34.4 MBq/mL in ethanol) in Ringer solution. Sections were washed 3 × 15 min with a Ringer solution and dipped briefly in distilled water at 4 °C. The slides were air-dried at 4 °C and used for autoradiography.

### 4.12. Radioactive Assays, Autoradiography, and Analysis

Air-dried brain sections were exposed for 5–10 days on tritium-sensitive phosphor imaging plates (BAS-IP TR2025, Fujifilm). Plates were scanned using the Fujifilm BAS-5000 phosphorimager and analyzed using MCID Analysis software (Imaging Research Inc, part of GE Healthcare). Regions of interest were selected, and bound radioactivity was analyzed based on a standard (American Radiolabeled Chemicals, ART0123, St. Louis, MO, USA) co-exposed with the brain sections. Bound radioactivity was expressed as ratios between the regions of interest in the transgenic animal divided by the bound radioactivity of the same regions in the wild-type control animal.

Dissected brain regions were weighted and homogenized in ice-cold homogenization buffer (1% NP-40, complete Ultra Proteinase Inhibitor tablets (Roche Diagnostics GmbH, Mannheim, Germany; according to manufacturer’s instructions) in 50 mM Tris/HCl, pH 7.4) using a Polytron PT1200 (Kinematica AG, Lucerne, Switzerland) and/or ultrasound (Branson Sonifier 250, Branson Ultraschall, Dietzenbach, Germany). A 100 µL aliquot was mixed with 3 mL UltimaGold scintillation cocktail (Perkin Elmer, Waltham, MA, USA) and incubated over night before beta counting in a TriCarb 2500 Beta Counter (Perkin Elmer Packard). Disintegrations per minute (DPM) were used to calculate concentrations of the radioactive tracer (fmol/mg) and %ID/g (percentage of injected dose per gram tissue weight).

## 5. Conclusions

This study explored the suitability of an antibody-based pretargeting approach for in vivo imaging of targets in the central nervous system. As a first step, the Aβ-specific, brain shuttle version of mAb31 (mAb31-BrainShuttle, short mAb) was successfully radiolabeled in vitro using the IEDDA reaction of a tritiated 1,2,4,5-tetrazine (Tz) group and a *trans*-cyclooctene modified mAb (mAb–TCO). The mAb–TCO–Tz antibody retained its functionality and specificities as demonstrated by in vitro experiments using PS2APP mouse brain sections, ELISA, FACS assays, radio-immunohistochemistry, and in vivo approaches. The radiolabeled Aβ-specific antibody crossed the blood-brain barrier and bound its target. One to three days after injection of mAb–TCO proved to be a suitable time window for in vivo pretargeting. The TCO groups remained reactive towards the tetrazine reactant since a specific radioactive labeling of Aβ plaques was evident in brain sections of PS2APP mice, using an ex vivo click reaction. However, the in vivo click reaction with intravenously injected Tz one and three days after injection of the TCO-modified antibody did not reveal a specific difference in binding intensity between the transgenic and WT animals. A radioactive signal was detected in brain tissue, but no click reaction was revealed by autoradiography. Further experiments are needed to optimize the multi-component pretargeting system, such as the TCO degree of labeling of the mAb, mode of conjugation of the TCO, concentration of Tz, and type of Tz. The right balance between stability and reactivity of both components can thus be observed for a successful in vivo pretargeting reaction. The presented study suggests that the application of PET imaging in drug development and for clinical diagnostics may be extended to targets currently considered undruggable and not approachable by a traditional small-molecule PET tracer.

## Figures and Tables

**Table 1:** Number of plaques detected by autoradiography after ex vivo click reaction. Data expressed as spots/mm^2^ (plaque density).

Plaque Density	1-Day Post-Injection		3-Days Post-Injection	
	PS2APP	WT	PS2APP	WT
Cortex	12.40 ± 2.13	3.63 ± 0.95	14.55 ± 3.27	3.30 ± 0.49
Hippocampus	12.12 ± 1.42	1.85 ± 0.47	11.84 ± 3.00	1.77 ± 0.44
Thalamus	9.45 ± 0.96	1.26 ± 0.40	9.15 ± 1.34	0.66 ± 0.18

**Table 2:** Study design and overview of in vivo experiments, used animals, and injected compounds.

Experiment	Animals Used		Antibody Injected In Vivo	Time (d) *	Tz Administered
	WT	PS2APP			
Brain Uptake		1×	mAb–TCO–Tz	3	in vitro, in vial
		2×	mAb–TCO–Tz	12	in vitro, in vial
In Vivo Stability of mAb–TCO	1×	1×	mAb–TCO	1	ex vivo, on slide
	1×	1×	mAb–TCO	3	ex vivo, on slide
	1×	1×	mAb–TCO	6	ex vivo, on slide
	1×	1×	mAb–TCO	12	ex vivo, on slide
In Vivo Click Reaction	2×	3×	mAb–TCO	1	in vivo, intravenous
	3×	3×	mAb–TCO	3	in vivo, intravenous

* Time interval post-injection of antibody to sacrifice animal or inject Tz.

## Data Availability

Data is contained within the article and the Supplementary Materials.

## Supplementary Materials

The following supporting information can be downloaded at: https://www.mdpi.com/article/10.3390/ph15121445/s1, Figure S1: Deconvoluted low-energy mass ([M+H]+) spectra of the main peaks of the light chains after reductive separation; Table S1: Assignment of masses found of the mAb light chain; Figure S2: Deconvoluted low-energy mass ([M+H]+) spectra of the main peaks of the heavy chain after reductive separation; Table S2: Assignment of the masses found of the mAb heavy chain; Figure S3: Deconvoluted low-energy mass ([M+H]+) spectra of the main peaks of the heavy chain + BrainShuttle module after reductive separation; Table S3: Assignment of the masses found of the mAb heavy chain + Brain Shuttle module; Figure S4: Immunohistochemistry of mAb and mAb-TCO in vitro on PS2APP brain sections; Table S4: Biodistribution data of radioactivity in PS2APP transgenic mice after in vivo injection of mAb-TCO-Tz; Figure S5: Immunohistochemistry analysis to mAb and mAb-TCO after in vivo injection in PS2APP transgenic mice; Table S5: Comparison binding ratios of mAb-TCO against mAb on PS2APP sections; Table S6: Single dose pharmacokinetic analysis of Tz in vivo behavior.
