# Supplementary material for: Antibody-Based In Vivo Imaging of Central Nervous System Targets—Evaluation of a Pretargeting Approach Utilizing a TCO-Conjugated Brain Shuttle Antibody and Radiolabeled Tetrazines
Source: Pharmaceuticals (Basel). 2022 Nov 22;15(12):1445. doi: 10.3390/ph15121445 (PMC9787164; doi:10.3390/ph15121445)
Supplement: Supplementary file 1 [file pharmaceuticals-15-01445-s001.zip › pharmaceuticals-1950085-supplementary.pdf]

pharmaceuticals

MDPI

Article

Supplemental Material to the manuscript:

Antibody-based In Vivo Imaging of Central Nervous System Targets – Evaluation of a Pretargeting Approach Utilizing a TCO-conjugated Brain Shuttle Antibody and Radiolabeled Tetrazines

Determination of Degree of Labeling

The chains (2x light chain, 1x heavy chain, 1x heavy chain with Brain Shuttle) were analyzed after partial reduction. The mass shifts of the light chains (Figure S1 and Table S1) as well as the heavy chain (Figure S2 and Table S2) were well assignable. For the heavy chain with the Brain Shuttle (Figure S3 and Table S3), the assignment was difficult. This is because there was a heterogeneous mixture of fragments in the sample. The *N*-terminal glutamine, for example, cyclized to pyroglutamic acid (PyroGlu), the protein was heterogeneously glycosylated, and there was also the varying degree of labeling. These are factors that make it difficult to accurately determine the degree of labeling. Deconvoluted analysis revealed a semi-quantitative assignment of 71% unlabeled and 39% 1x label for the light chains; for the heavy chain: 39% unlabeled and 61% 1x label; for the heavy chain with Brain Shuttle: 9% unlabeled, 45% 1x label, and 46% 2x label. Using the Poison distribution, this results in an average degree of labeling of 2.6 labels per protein.

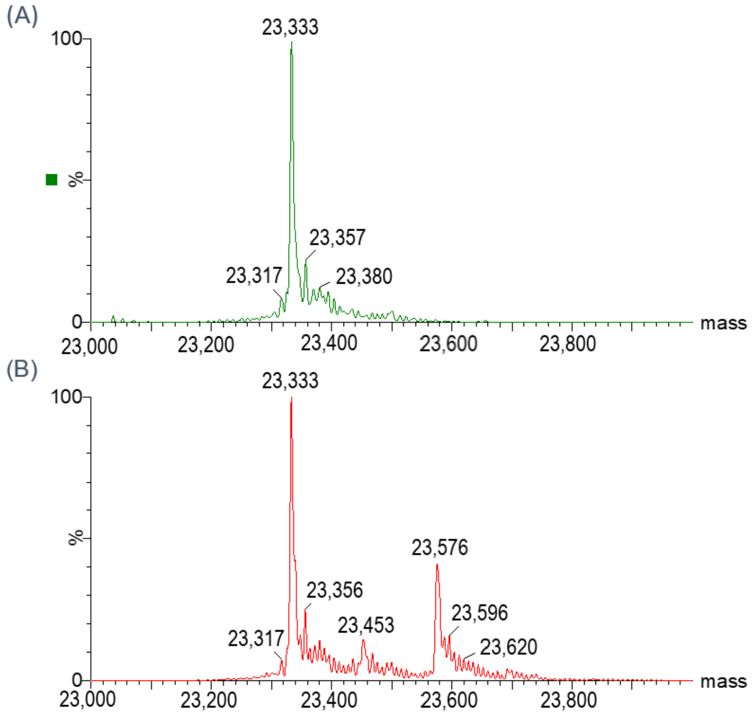

Figure S1. Deconvoluted low-energy mass ([M+H]+) spectra of the main peaks of the light chains after reductive separation. (**A**) Spectra of the unlabeled sample. (**B**) Spectra of the TCO-labeled sample.

Table S1. Assignment of masses found of the mAb light chain.

Mass found [Da]	Suggestion	Theoretical mass [Da]	Mass error [Da]	Relative amount
**Unlabeled**				
23,333	Light Chain	23,339	-6	100%
**TCO-labeled**				
23,333	Light Chain	23,339	-6	71%
23,576	1x labeled Light Chain	23,581	-5	29%

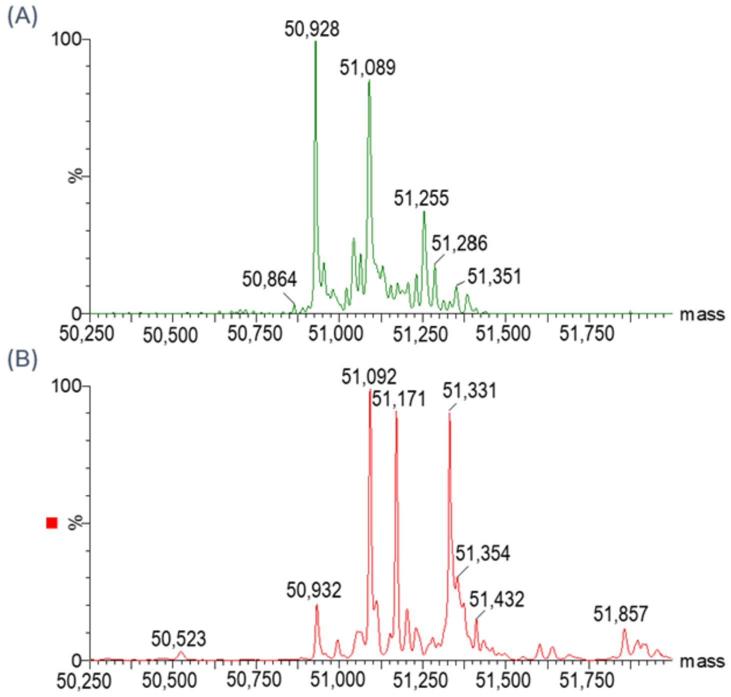

Figure S2. Deconvoluted low-energy mass ([M+H]+) spectra of the main peaks of the heavy chain after reductive separation. (**A**) Spectra of the unlabeled sample. (**B**) Spectra of the TCO-labeled sample.

Table S2. Assignment of the masses found of the mAb heavy chain.

Mass found [Da]	Suggestion	Theoretical mass [Da]	Mass error [Da]	Relative amount
**Unlabeled**				
50,928	PyroGlu HC (hole) LysCut +G0F	50,935	-7 Da	100%
51,089	PyroGlu HC (hole) LysCut +G1F	51,098	-9 Da	
**TCO-labeled**				
50,932	PyroGlu HC (hole) LysCut +G0F	50,935	-3 Da	39%
51,092	PyroGlu HC (hole) LysCut +G1F	51,098	-6 Da	
51,171	1x labeled PyroGlu HC (hole) LysCut +G0F	51,170	-1 Da	61%
51,331	1x labeled PyroGlu HC (hole) LysCut +G1F	51,340	-9 Da	

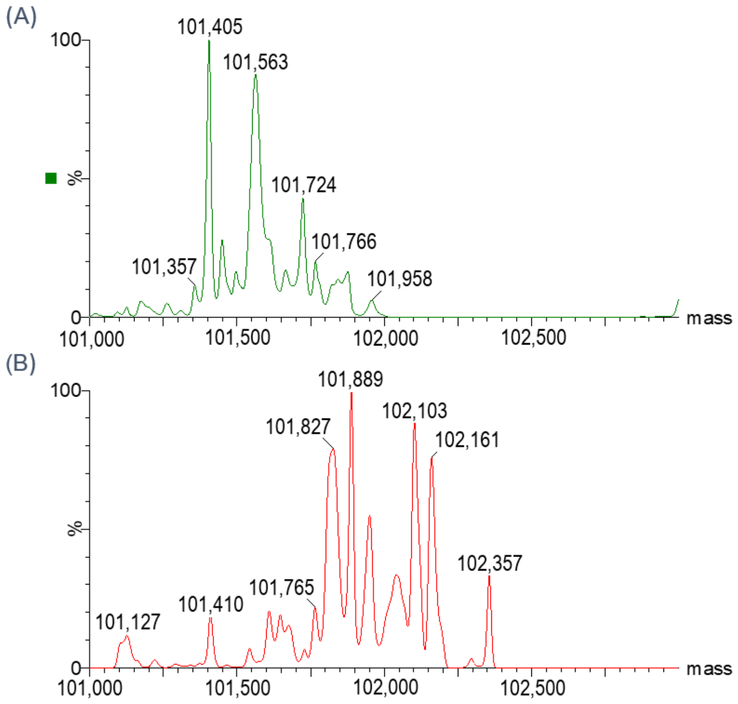

Figure S3. Deconvoluted low-energy mass ([M+H]+) spectra of the main peaks of the heavy chain + BrainShuttle module after reductive separation. (**A**) Spectra of the unlabeled sample. (**B**) Spectra of the TCO-labeled sample.

Table S3. Assignment of the masses found of the mAb heavy chain + Brain Shuttle module.

Mass found [Da]	Suggestion	Theoretical mass [Da]	Mass error [Da]	Relative amount
**Unlabeled**				
101,405	PyroGlu HC (knob) +G0F	101,415	-10 Da	100%
101,563	PyroGlu HC (knob) +G1F	101,578	-15 Da	
**TCO-labeled**				
101,410	PyroGlu HC (knob) +G0F	101,415	-5 Da	9%
Not detected	PyroGlu HC (knob) +G1F	101,578	-	
101,647	1x labeled PyroGlu HC (knob) +G0F	101,657	-10 Da	45%
101,827	1x labeled PyroGlu HC (knob) +G1F	101,820	+7 Da	
101,889	2x labeled PyroGlu HC (knob) +G0F	101,900	-11 Da	46%
Not detected	2x labeled PyroGlu HC (knob) +G1F	102,063	-	

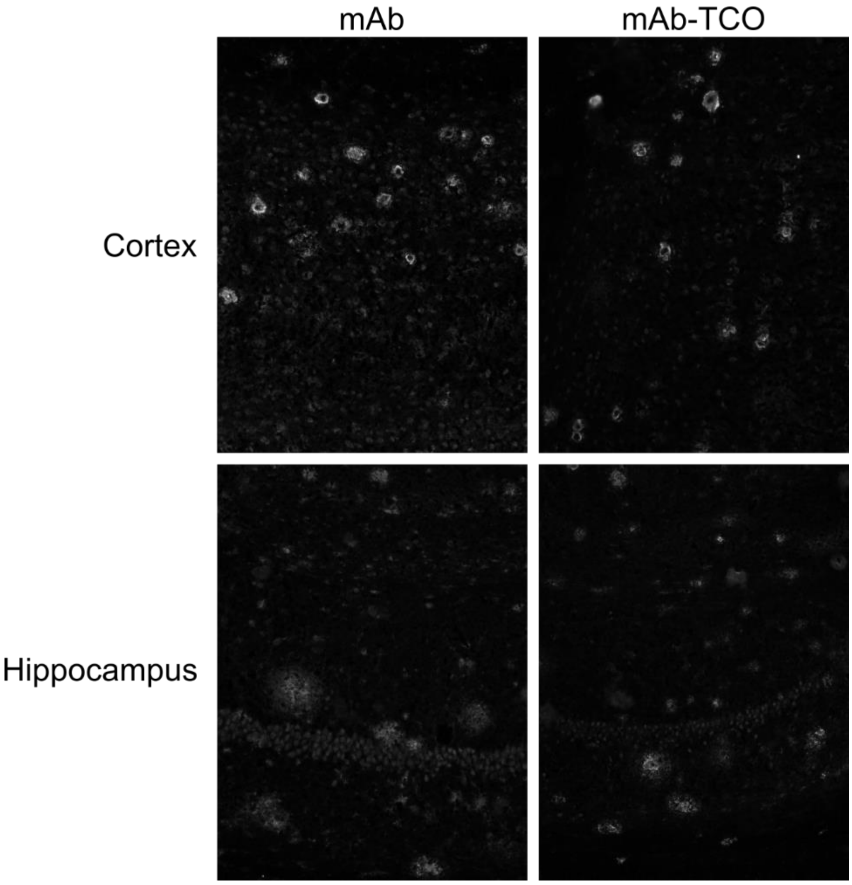

Figure S4. Immunohistochemistry of mAb and mAb-TCO in vitro on PS2APP brain sections. Brain sections of PS2APP transgenic mice were incubated with 1ug/ml of mAb and mAb-TCO solution, respectively. The staining was visualized with a fluorescently labeled secondary goat-anti-human antibody. Examples the cortex and hippocampus from light microscopic images (10x magnification) are presented in the figure.

Table S4. Biodistribution data of radioactivity in PS2APP transgenic mice after in vivo injection of mAb-TCO-Tz.

Brain region/organ	1 d	3 d	6 d	12 d
	[%iD/g]			
Serum	1.34	0.49	0.25	0.01
Cerebellum	0.45	0.33	0.19	0.09
Cortex	0.56	0.51	0.33	0.21
Hippocampus	0.56	0.51	0.34	0.19
Rest Brain	0.47	0.45	0.26	0.15
Liver	1.85	0.80	0.39	0.13
Kidney	0.92	0.47	0.23	0.07

The biodistribution data of mAb-TCO-Tz clearly demonstrate, that the antibody construct enters the brain an accumulates at higher concentrations in brain regions with pronounced Aβ plaque pathology like the cortex or hippocampus. However, it also shows the relatively long persistence of the mAb-TCO-Tz in the periphery of the body, as demonstrated by the remaining radioactive signal in the serum and other peripheral organs like the liver and kidney (two main routes of excretion of mAbs).

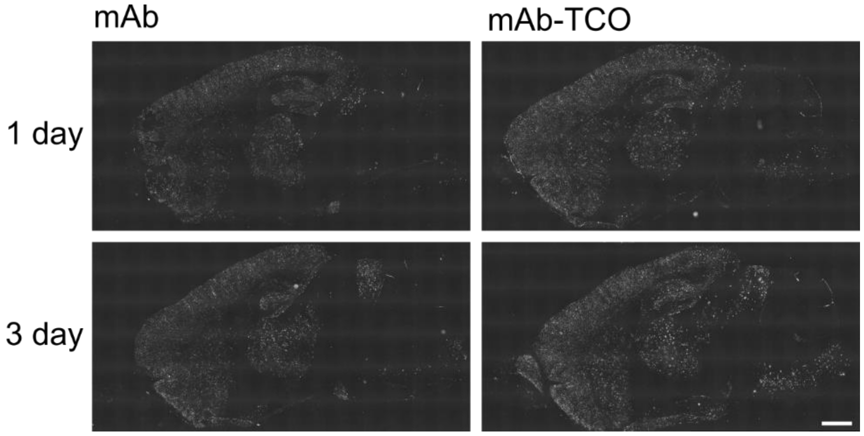

Figure S5. Immunohistochemistry analysis to mAb and mAb-TCO after in vivo injection in PS2APP transgenic mice. 20mg/kg antibody construct were injected i.v. in PS2APP transgenic animals. Animals were sacrificed and the brain was dissected after 1 and 3 days. Sagittal brain sections were stained with a goat-anti-human fluorescently labeled antibody to detect the injected mAb and mAb-TCO construct.

Table S5. Comparison binding ratios of mAb-TCO against mAb on PS2APP sections.

mAb-TCO + Tz			
Brain region	in vitro	ex vivo 1 day	ex vivo 3 days
Cerebellum	3.90	0.98	1.04
Cortex	6.66	1.04	1.22
Hippocampus	4.56	1.04	1.21
Thalamus	2.81	1.17	1.19

Comparison of binding ratios of the radioactively signal determined by Autoradiography on PS2APP brain sections. In vitro: PS2APP brain sections were incubated with 1µg/mL mAb-TCO or mAb in vitro followed by an on slice click reaction with 100 nM Tz for 2 h at 22 °C. The ratio was determined as the radioactive signal on mAb-TCO divided by the signal on the corresponding brain region from PS2APP section incubated with the mAb.
Ex vivo: PS2APP animals were injected with 20 mg/kg mAb-TCO or mAb. After 1 and 3 days, the animals were sacrificed, and sagittal brain sections were incubated with 100 nM Tz for 2 h at 22 °C. Again, the ratio was calculated by the radioactive signal in the mAb-TCO sections divided by the signal from the corresponding brain regions from animals injected with the mAb construct.

Table S6. Single dose pharmacokinetic analysis of Tz in vivo behavior.

	2	5	15	30	120	240	1440
				[min]			
Organ/Tissue				%iD/g			
Plasma	5.44	4.77	3.81	3.14	3.14	3.01	2.41
Cerebellum	3.22	2.60	2.03	1.70	1.93	1.80	1.56
Rest Brain	3.02	2.69	2.04	1.70	1.92	1.94	1.68
Lung	4.27	5.03	3.87	2.62	3.10	3.16	1.62
Liver	11.19	11.84	13.27	12.96	12.34	9.96	3.06
Kidney	7.40	9.51	7.78	4.17	3.20	2.59	2.17
Bladder (empty)	4.42	7.46	14.66	7.44	5.51	5.81	1.96
Small Intestine (excl. content)	3.62	4.48	4.69	3.75	2.75	2.59	2.05
Fat	0.66	0.96	1.02	0.46	0.45	0.54	0.32
Muscle	3.12	3.40	2.37	2.02	1.87	1.83	1.40
Tail	7.57	2.19	2.75	3.44	1.69	1.58	1.40

48.1 MBq/kg Tz in PBS were injected *i. v.* in wildtype mice. Animals were decapitated after indicated timepoints, blood was sampled, and organs dissected. The biodistribution data of Tz clearly demonstrate that the compound enters the brain quickly after i.v. injection. However, it also shows the relatively long persistence of the Tz in the periphery of the body, as demonstrated by the remaining radioactive signal in the serum and other peripheral organs like the liver, kidney, or bladder (major routes of excretion of the mAbs).
